# Winter condition variability decreases the economic sustainability of reindeer husbandry

**DOI:** 10.1002/eap.2719

**Published:** 2022-11-15

**Authors:** Antti‐Juhani Pekkarinen, Sirpa Rasmus, Jouko Kumpula, Olli Tahvonen

**Affiliations:** ^1^ Natural Resources Institute Finland Helsinki Finland; ^2^ Department of Forest Sciences University of Helsinki Helsinki Finland; ^3^ Arctic Centre University of Lapland Rovaniemi Finland; ^4^ Natural Resources Institute Finland Kaamanen Finland

**Keywords:** adaptation to climate change, bioeconomic modeling, economic sustainability, practitioner knowledge, reindeer (*Rangifer tarandus* tarandus), reindeer husbandry, snow conditions, supplementary feeding, winter weather

## Abstract

Wild and semidomesticated reindeer are one of the key species in Arctic and subarctic areas, and their population dynamics are closely tied to winter conditions. Difficult snow conditions have been found to decrease the calving success and survivability of reindeer, but the economic effects of variation in winter conditions on reindeer husbandry have not been studied. In this study, we combine state‐of‐the‐art economic–ecological modeling with the analysis of annual reindeer management reports from Finland. These contain local knowledge of herding communities. We quantify the occurrence probabilities of different types of winters from annual management reports and analyze the effects of this variation in winter conditions on reindeer husbandry using an age‐ and sex‐structured bioeconomic reindeer–lichen model. Our results show that difficult winters decrease the net revenues of reindeer husbandry. However, they also protect lichen pastures from grazing, thereby increasing future net revenues. Nonetheless, our solutions show that the variability of winter conditions overall decrease the net income of herders compared to constant winter conditions. Low lichen biomass appears to make reindeer management more sensitive to the effects of difficult winter conditions. We also found that it is economically sensible to use supplementary feeding during difficult winters, but the net revenues still decrease compared to average winters because of the high feeding costs. Overall, our analysis suggests that the increasing variability of winter conditions due to climate change will decrease net revenues in reindeer husbandry. This decrease will still occur even if the most extreme effects of climate change do not occur. This study shows that combining a state‐of‐the‐art bioeconomic model and practitioner knowledge can bring compatible insights, ideas, results, and a bottom‐up perspective to the discussion.

## INTRODUCTION

Ungulate life history strategies in Arctic and subarctic regions are determined by seasonality and winter food limitation (Danell et al., 2006). Besides population dynamics, the variation in winter conditions also affects the economically sustainable harvesting of ungulate populations. Wild and semidomesticated reindeer are one of the key species in Arctic and subarctic areas (Forbes et al., 2006; Uboni et al., 2016) and a prime example of an Arctic ungulate with population dynamics closely tied to winter conditions (Fauchald et al., 2004; Helle & Kojola, 2008; Kumpula & Colpaert, 2003). Reindeer husbandry is also an integral part of the social and economic life of many northern indigenous and local communities. It represents cultural continuity and a way of life; it also employs people, keeps remote communities inhabited, and provides regional economic benefits (Holand et al., 2022).

However, most of the research on reindeer focuses on the biology and ecology of reindeer, so interdisciplinary approaches are needed for studying this complex system (Pape & Löffler, 2012). One suitable approach is the use of economic–ecological models for studying reindeer herding economy and optimal herding strategies (Pekkarinen, 2018). In addition, practitioner knowledge can provide valuable insights on the properties of the system and incorporate the perspective of herder knowledge into the discussion. Thus, in this study, we combined the analysis of annual reports of reindeer herding districts with an economic–ecological optimization model of a reindeer husbandry system. Our aim was to study how the typical between‐year variation of winter conditions and potential increase of this variation due to climate change affected the economic sustainability of reindeer husbandry.

The conditions and availability of winter forage are limiting factors for the survival, growth, and productivity rates of reindeer populations in many parts of Fennoscandia (Kojola et al., 1995; Kumpula & Colpaert, 2003; Tveraa et al., 2003). Winter conditions determine the energy balance of reindeer, consequently affecting the number of calves born the next spring and the survivability and productivity of the reindeer population as a whole (Albon et al., 2002; Tveraa et al., 2003). The depth, hardness, and compactness of the snow and icing of ground snow on the vegetation interface affect the cratering conditions through the snow cover, energy needs, and energy intake of reindeer (Heggberget et al., 2002; Kumpula, 2001). Lichen pastures are an especially important energy source during winter. When winter conditions are difficult and reindeer are unable to satisfy their energy needs, their spring weight decreases, and calf production, calf size, and adult survivability are all lower (Tveraa et al., 2003). In contrast, with easily cratering conditions and very high resource availability, reindeer can conserve or even build up body reserves as a precaution against possible upcoming difficult conditions (Bårdsen et al., 2009). When lichen availability is high, lichen consumption by reindeer may exceed their energy needs (Nieminen et al., 1987), which, together with more protein‐rich food (dwarf shrubs, sedges, and hays), allows reindeer to remain in good physical health, and even increase their weight during winter. However, this extra energy intake and weight gain during winter seem to have little or no effect on the reproductive output of reindeer or on the summer weight of adults beyond the normal upper limit (Bårdsen et al., 2009; Fauchald et al., 2004). This overuse of pasture resources is especially evident when lichen availability is high (Nieminen et al., 1987).

Although previous studies found that difficult winters decreased the productivity of a reindeer population, they did not examine the consequences of winter condition variability on the economics of reindeer husbandry or on sustainable harvesting and population levels. In this study, we analyzed how changes in winter conditions affected the economics of reindeer husbandry. We investigated the effects of a single difficult or easy winter, along with the long‐term effects of the typical variation in winter conditions (excluding exceptional conditions). We combined economic–ecological model analysis with practitioner knowledge. For economic–ecological model analysis, we used the bioeconomic model by Tahvonen et al. (2014) and Pekkarinen et al. (2015). Wintertime resource availability is the main factor for reindeer population dynamics in this model. In addition, it includes the nonlinear effects of winter energy accumulation (decreasing weight, survivability, reproduction during difficult winters, and the overuse of pasture resources during easy winters). These features make the model suitable for studying the economics of reindeer husbandry under varying winter conditions. We combine the analysis of this bioeconomic model with an analysis of annual management reports from reindeer herding districts. These management reports incorporate local traditional knowledge of herding communities. They reflect authentic, contemporary voices of reindeer herders and their perspectives concerning difficult winter conditions and consequent effects on herding.

By analyzing the annual reports, we answer the following questions: (1) How often have difficult winters occurred in northernmost Finnish Lapland during the period 1981–2010? (2) What are the impacts of difficult winter conditions on reindeer populations and herding practices? Then, using the bioeconomic reindeer–lichen model, we investigate (3) how varying winter conditions affect the economically optimal model solutions and (4) the costs of increasing variation in winter conditions for reindeer husbandry. Finally, we discuss our bioeconomic modeling solutions using excerpts from the annual reports, bringing a bottom‐up perspective to the discussion.

## DATA, MODEL, AND METHODS

### Analysis of annual herding district reports

Our study area covers the 20 northernmost herding districts of the reindeer management area in Finland. Finland has 54 herding districts, and the organization and activities of these districts are guided by legislation (Reindeer Husbandry Act, 848/1990). District size varies along with the maximum number of animals that reindeer owners in the district are allowed to keep. In 2019, there were 4354 reindeer owners in Finland, and the total maximum allowed size of the winter stock (number of adult animals left alive in the autumn round‐ups, number of adult animals grazing within an area during winter) is 203,700 reindeer. During 2018–2019, the size of the winter stock was 188,190 animals. Annually, approximately 85,000–100,000 calves are born in the spring and the same number of calves and adults are slaughtered during autumn.

Herding districts are required to compile an annual management report for the Reindeer Herders Association (RHA) after each herding year (which begins 1 June and ends 31 May). Several name changes, mergers, and subdivisions of districts have taken place over the decades. However, these changes were tracked during data analysis, and present‐day district subdivisions were used as a baseline for the whole study period. Reports from 1982 onward are kept in the RHA archives in Rovaniemi, and earlier reports are kept in the National Archives in Oulu.

Using annual reports, we listed each reference mentioning difficult winter conditions and their effects on the reindeer population and herding practices. We counted all the references where winters were considered more difficult than average. We also examined the reported causes for the difficult winter conditions (e.g., deep snow, icy snow). In addition, we analyzed how often winter conditions reportedly caused decreased calving success or increased mortality or the need for supplementary feeding. In this study, we were particularly interested in winters that reportedly decreased calving success or increased mortality due to deep or icy snow, because such effects would be included in the bioeconomic model. The analysis was qualitative (based on written descriptions found in the reports). The reports do not contain economic information (funds used, income data) that would enable quantitatively analyzing, for example, the outcome of certain coping strategies. For the analysis, we used reports from 1981 to 2010, because this is the current climatological standard period (http://www.wmo.int/pages/prog/wcp/wcdmp/GCDS_1.php). We additionally present some excerpts from this material to illustrate and discuss the themes of the article from a practitioner viewpoint. The temporal and spatial coverage of these excerpts is larger than the 20 northernmost herding districts (i.e., they cover all herding districts in Finland and earlier herding years).

Management reports have seldom been used in research. The reliability of the reports is somewhat diminished by differing reporting styles (among individuals, between decades and districts). Data may be missing due to inexact note taking in some districts during some years (empty reports or missing references for certain subjects; only a few reports are entirely missing per year). Nevertheless, these reports are valuable historical material. They represent authentic, contemporary voices of reindeer herders and their perspectives concerning difficult winter conditions and their subsequent effects on herding. We consider this to be local knowledge or practitioner knowledge (Forbes et al., 2019; Ingold, 2000), which is a valuable source along with scientific knowledge. Herders possess knowledge concerning the natural conditions within their own herding districts, and this knowledge has in many cases been accumulating since childhood and is commonly reflected in local practices (Forbes, 2006; Helander‐Renvall, 2014).

By analyzing the practitioner knowledge found in the annual reports, we gain more in‐depth understanding about the natural conditions within the studied 20 northernmost herding districts compared to using a modeling approach alone. Widening the analysis to include other herding districts and over a longer time perspective further enriches the discussion concerning optimization outputs with aspects such as impacts on livelihood, the combined effects of weather and other land uses, and coping.

### Bioeconomic model of reindeer husbandry

In addition to analyzing the annual reports, we used a bioeconomic model of the reindeer husbandry system to study the effects of varying winter conditions. Bioeconomics is the study of economically optimal renewable resource use, and it typically utilizes economic–ecological models of the subject system (Clark, 1990). In this study, we expanded the bioeconomic model presented in detail in Pekkarinen et al. (2015) by including the effects of varying winter conditions. The reindeer population model includes 17 female and 13 male age classes and a detailed description of winter energy resource utilization. Reproduction is specified by a modified harmonic mean mating system (Bessa‐Gomes et al., 2010), and diet choice follows the principles of the optimal foraging theory (e.g., Stephens, 1986). Winter food availability and the associated energy intake in relation to wintertime energy needs define an individual's weight decrease and its consequences for mortality and reproduction. Lichen growth depends on the areas of lichen‐dominated habitat types and their lichen biomass after consumption. The decision variables are the animals chosen for slaughter from the age and sex classes and the amount of supplementary food given. The mathematical description of the model is presented in Appendix S1, and the model code is available for download (Pekkarinen, Tahvonen, & Kumpula, 2022).

The structure of this model is suitable for studying economically optimal reindeer husbandry under varying winter conditions because it assumes that summer pastures are sufficient, and thus wintertime resource availability is the main factor influencing reindeer population dynamics. In addition, it includes a description of the nonlinear relation between winter energy accumulation and population growth rates. Thus, the model takes into account that reindeer are unable to properly satisfy their energy needs under low resource availability. This leads to decreased spring weight, calf production, calf size, and adult survivability (Tveraa et al., 2003). In contrast, very high resource availability causes increased consumption during winter but does not benefit calf production or survivability beyond the normal upper limit during average or easy winters. Thus, an excess of winter resources (lichen or supplementary forage) leads reindeer to consume more energy, which may increase their spring weights (Fauchald et al., 2004). However, this overuse of pasture resources does not benefit calf production and survivability beyond the normal upper limit. Also, slaughter weights during the following autumn are unaffected by excess resource use or excess weight gain during the previous winter (Bårdsen et al., 2009).

The excess energy intake during early or midwinter acts as an insurance against possible difficult conditions during late winter (Bårdsen et al., 2009). However, if food conditions remain easy throughout the winter, this excess energy intake during early or midwinter does not provide any extra benefit for reindeer or reindeer husbandry. On the contrary, it disadvantages reindeer husbandry because pasture overuse entails the consumption of valuable winter energy resources (natural pastures or supplementary food) beyond what would have been needed to ensure an optimal production rate of the population. This nonlinear relation between excess resource use during winter and population growth rate is typically forgotten in age‐structured models used for studying sustainable harvesting strategies (Lande et al., 2003). However, in the economically optimal management solutions presented in the Tahvonen et al. (2014) and Pekkarinen et al. (2015) models, this nonlinear relation clearly influences the optimal size and structure of reindeer populations, optimal lichen biomass, and optimal use of Supplementary food.

Ground lichens and other cratered food items, including dwarf shrubs, hays, and sedges, are natural winter energy resources used in the model version. The annual quantity of supplementary food is optimized separately for each year. When supplementary food is provided, reindeer utilize a mixed diet that includes ground lichens, other cratered food items, and supplementary food. In the model, the choice between various energy resources is driven by the availabilities of these resources. Reindeer prefer lichen whenever available, and therefore more than 80% of their energy is acquired from lichens when lichen biomass is high (>1000 kg/ha). Other cratered resources become more important when lichen availability is low. With very low lichen biomass (<300 kg/ha), most of the energy comes from other cratered food items and, if available, from Supplementary food.

### Restrictions of model version used in this study

The model in Pekkarinen et al. (2015) includes the effect of various lichen pasture types and pasture conditions on lichen availability and growth. In addition, it can also be used for studying the effects of pasture rotation and supplementary feeding on reindeer husbandry. In this study, we assumed that a closed pasture rotation system was used, that is, the winter lichen pastures are consumed only during winter. To keep the analysis simple, we computed all the solutions for a hypothetical herding district, where lichen pastures are located in old‐growth or mature pine forests, so the ground lichen growth rate is high. To further simplify the analysis, we did not consider the effects of arboreal lichen pastures in this study.

The bioeconomic reindeer husbandry model has also been further developed to include the effects of predation (Pekkarinen et al., 2020) and various winter pasture conditions (Pekkarinen et al., 2021). However, in this study, we used a model version that only included ground lichens and other cratered food items as natural winter energy resources as well as the possibility of supplementary feeding. We did not include the effects of predation, arboreal lichens, or the regional variation in growth conditions and pasture rotation systems. In addition, we did not vary herding costs, feeding costs, slaughtering costs, meat prices, or government subsidy systems. All these factors were examined with deterministic model versions from previous studies (Pekkarinen et al., 2015, 2020, 2021; Pekkarinen, Kumpula, et al., 2022).

**FIGURE 1:**
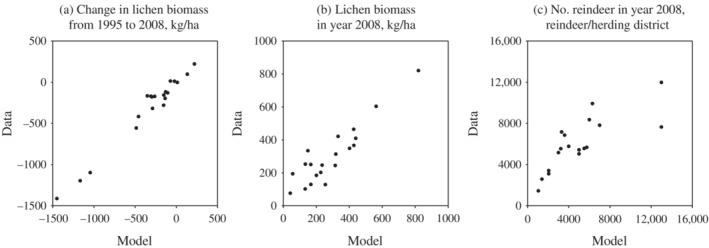
Model solutions compared with data from 20 northernmost reindeer herding districts in Finland. Figures are originally presented in Pekkarinen et al. (2017) and redrawn using the original data sets. (a, b) Measured data points compared with model predictions. (c) Comparison of observed reindeer numbers with economically optimal model solutions.

In the model version used in this study, system dynamics are based on reindeer–lichen interactions and supplementary feeding. Pekkarinen et al. (2017) showed that the bioeconomic reindeer husbandry model could describe these interactions and the balance between reindeer populations and lichen biomass. Figure 1 is a reillustration of the solutions found in Pekkarinen et al. (2017). Figure 1a,b shows that the model performs well in predicting the lichen biomass and how much this biomass has changed due to reindeer consumption. Figure 1c shows that the actual reindeer numbers in Finnish herding districts are close to optimal model solutions. Although these model solutions consider the effects of regional differences in herding conditions, they present economically optimal model solutions, whereas actual reindeer numbers may also be affected by other incentives that herders may have. In addition, actual reindeer numbers are restricted by the maximum number of reindeer allowed by the Ministry of Agriculture and Forestry of Finland.

Regional differences were not included in this study, which concentrated on the general underlying dynamics between the reindeer population and lichen pastures and their economically optimal utilization. Our main aim was to examine how varying winter conditions affected the economically optimal management of the reindeer–lichen system. However, because the model included the age and sex structures of the reindeer population, it could also be used to study the optimal slaughtering strategy. We therefore also investigated how well the optimal slaughtering strategy coincided with the actual slaughtering strategy used by the 20 northernmost herding districts in Finland during the previous climatological standard period, 1981–2010. Data from the districts were originally published by the RHA in *Poromies* magazine. Reindeer herding statistics from each Finnish reindeer herding district are published annually by Reindeer Herders Association in *Poromies* magazines second number of each year. This study uses data published in *Poromies* magazines second numbers during years 1982–2011.

### Defining the probabilities of difficult winters for the bioeconomic model

Winter conditions can be more difficult or easy for reindeer herding during some years due to various reasons (e.g., deep, hard, and compact snow, snow covering ice, extreme temperatures, late snowmelt) compared to average conditions. In this study, we defined a difficult winter as a winter with deeper, harder, or more compact snow cover, thicker ice layers within the snow cover, or more extensive or long‐term icing of lichens below the snow compared to average winters. These snow characteristics reduce the cratering area of the reindeer (Kumpula, 2001; Kumpula et al., 2004) and increase their energy needs (Fancy & White, 1985). Similarly, cratering is easier during easy winters, and energy needs are lower due to easier snow and icing conditions.

Changes in cratering conditions and energy needs in turn affect the body condition of reindeer and consequently also to the number and weight of newborn calves along with adult wintertime mortality. For bioeconomic model computations, we divide winter difficulty into three categories: easy, normal, and difficult. We first study the effects of a single difficult or easy winter and then the effects of winter condition stochasticity.

**FIGURE 2:**
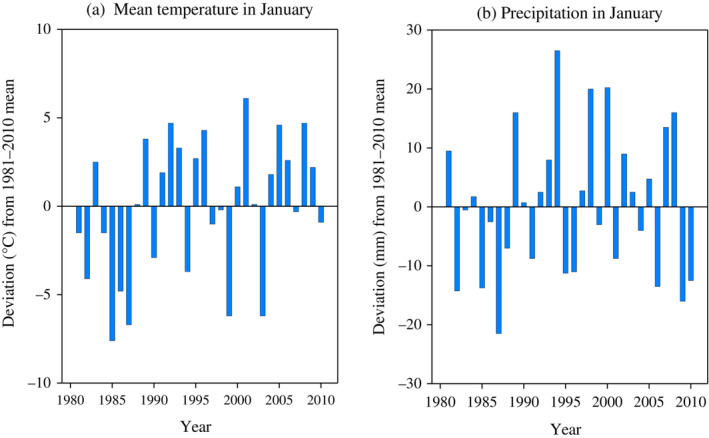
Mean temperature and precipitation in January at Inari weather station in northernmost Finnish Lapland. Panels present annual deviation from long‐term (1981–2010) average mean temperature (−12.7°C) and average precipitation (25 mm) in January.

We estimated the probability of difficult winters from previous research and compared it with the analysis contained in annual reports. To study how the stochasticity of winter conditions alone affected the profitability of reindeer husbandry, we assumed that winter difficulty remained at the same level on average. Thus, we assumed that the probabilities of different winter types were symmetric, that is, easy winters occurred with the same probability as difficult ones (the remaining winters were defined as normal or average). The assumption of symmetricity was also supported by the statistics of the Finnish Meteorological Institute. For example, they showed that temperature and precipitation values in January were rather evenly distributed around the mean during our study period (1981–2010) (Figure 2a,b). The January temperature was below the mean for 14 years out of the 30‐year period and above the mean for 16 years. Similarly, precipitation has been below average for 15 of the study years and above average for 15 years.

According to Helle and Kojola (2008), difficult winters (severe icing of lichen pastures) have occurred once every 7 years on average in the Finnish reindeer management area. Rasmus et al. (2014) found the occurrence of difficult winter conditions to be roughly once per decade. Thus, for the bioeconomic modeling computations, we used a 10% probability for difficult winters because this was supported by the results of the previous studies and also coincided with the general definitions of a rare event (IPCC, 2013). However, we also used annual reports to study and define the probability of difficult winters from a herder's perspective. We then compared that with the approximately 10% probability found in previous research. Because we used a symmetric distribution, the probability for easy winters was also 10%. These probabilities can be viewed as an approximation of the occurrence frequencies of different winter conditions in northernmost Finnish Lapland in recent decades.

To study the economics of reindeer husbandry under this typical variation in winter conditions, we used a random number generator to produce the realizations of these winter conditions from the defined probabilities. However, we also studied how the increasing probability of easy and difficult winters affects the economics of reindeer herding. Climate change has been estimated to increase the variation in winter conditions in both amplitude and frequency. In this study, we only examined the effect of the increasing variation frequency under different winter conditions on the reindeer herding economy. In addition, we limited our analysis to the observed typical between‐year variation of winter conditions. Thus, we excluded the effects of extreme conditions that might occur a few times per century and cause atypically severe reindeer losses.

### Defining the effect of difficult winter conditions on reindeer energy needs

Difficult winters affect the energy balance of reindeer in two ways: first, by raising cratering and movement costs, and second, by decreasing their energy intake via a reduced cratering area. Boertje (1985) modeled the energy requirements of female caribou and found that difficult cratering conditions could increase energy expenditure by approximately 5%. However, Gotaas et al. (2000) found that the factorial models were too conservative for assessing total energy needs and that the energy needed for locomotion in particular varied more than previously expected. Fancy and White (1985) studied the increase in reindeer energy expenditure when cratering through thinly crusted snow. They found that the net hourly energy expenditures in uncrusted and thinly crusted snow were 1.2–1.5 kJ/kg and 2.3–2.9 kJ/kg, respectively. Using these estimates, the energy needs of female reindeer (ca. 18 MJ/winter day) and assuming an 8‐h cratering time results in a 6% energy need increase for adult reindeer when cratering in thinly crusted snow. Thus, in our bioeconomic model, we assumed that the energy need was 6% higher during difficult winters and 6% lower during easy winters compared to normal winters (see Appendix S1 for the mathematical description of how increasing energy need is included in the model). During extremely difficult winters, the energy need may increase even more, but in this study, we only considered the typical variation in winter conditions, not the most extreme years.

### Defining the effect of difficult winter conditions on the cratering area of reindeer

The daily cratering area of reindeer in winter varies considerably, depending on snow conditions and lichen availability (Kumpula, 2001; Kumpula et al., 2004). In their bioeconomic model, Pekkarinen et al. (2015) used an average cratering area of 30 m^2^/day, which has been found to be a suitable average for typical winters in coniferous areas in northernmost Finnish Lapland (Kumpula, 2001). Although cratering area has been found to decrease during difficult winters (Collins & Smith, 1991; Kumpula, 2001; Pruitt, 1992), we are unaware of any studies examining how much the cratering area decreases. However, using reindeer husbandry statistics and our model setup, we can produce a rough estimate on how much the cratering area decreases during difficult winters. According to Finnish reindeer husbandry statistics, the average calf percentage in the 20 northernmost herding districts was 52% during 1960–2016 and the lowest decile of calf percentage was 30% (statistics of the RHA). Thus, during average difficult years (excluding extremely difficult years), calf percentage decreased by roughly 20 percentage points. This corresponds to a 4‐m^2^ reduction in daily cratering area in our model. With a 30‐m^2^ cratering area and a lichen biomass of 500 kg/ha, our model produced a 52% calf percentage during average winters, and assuming a 4‐m^2^ reduction for this cratering area produced a 30% calf percentage. Thus, using the 4‐m^2^ decrease in cratering area to describe the cratering conditions during difficult winters led to a relatively good description of the typical variation in calf percentages found in Finnish reindeer herding area in recent decades. Because we used symmetric variation in winter conditions, we assumed that during easy winters the cratering area increased by 4 m^2^. Thus, in bioeconomic modeling, average daily cratering areas of 34, 30, and 26 m^2^ are used for easy, normal, and difficult winters (see Appendix S1 for a mathematical description of how the change in cratering area was included in the model).

Deep and hard snow cover decreases the total cratering area and number of craters (Collins & Smith, 1991) and, thus, decreases lichen consumption. Reindeer may even stop cratering altogether in very deep and hard snow conditions (Kumpula, 2001). In the model used in this study, the decreasing cratering area decreased the daily energy intake from ground lichens and other cratered food items. When lichen biomass is high, reindeer can compensate the reduced cratering speed by increasing their cratering time. However, this compensation is not possible with lower lichen biomass, because a reindeer needs all the available cratering time to meet its energy needs even during normal winter conditions. In this case, the energy intake from lichen and lichen consumption decrease during difficult winter conditions as the cratering area decreases. Thus, difficult cratering conditions cause reindeer to lose weight but simultaneously protect lichen pastures from consumption and wastage.

### Economic optimization

We used Knitro optimization software (versions 7.0 and 10.3) and AMPL programming language (Byrd et al., 2006) for all computations and optimizations. To study the effects of interest rate on the economics of reindeer herding in varying winter conditions, we computed the dynamic solutions with different levels (from 0% to 5%) of annual interest rates. The interest rate describes the expected rate of return from alternative investment possibilities. Similarly, it can be viewed as a time preference for current over future income.

To study stochastic winter conditions, we assumed that 10% of the winters were difficult, 10% were easy, and 80% were average. We repeated the computations 50 times with different scenarios of stochastic winter conditions to compute the average present value of net revenues between computations. We recognized that 50 repeats were not enough to fully examine the possible outcomes of these stochastic events. However, because optimization computation requires a lot of time, we felt that 50 repeats was a reasonable trade‐off and provided enough insight on system properties.

We computed the dynamic solutions with a time horizon of 100–150 years, which was sufficient to produce a good approximation of the steady‐state long‐term sustainable solution. In dynamic optimization with varying winter conditions, we computed optimal deterministic feedback solutions. In deterministic feedback solutions, optimization is repeated at the beginning of each time period (model year begins in autumn after slaughtering) and the system state is updated the following year based on these optimizations. Thus, the herding district observes and reacts to the emerging information on reindeer population and pasture conditions by reoptimizing the slaughtering and feeding strategies at the beginning of each herding year. This optimization has an infinitely long time horizon, but the degree of difficulty of future winters is based on expected conditions. This is an example of applying the certainty equivalence principle instead of, for example, stochastic dynamic programming.

Applying stochastic optimization would be beyond our computational capabilities because of the “curse of dimensionality,” that is, stochastic dynamic programming is normally possible only for models with a low number of state variables (<5). Simplifying a model's ecological properties could allow the use of stochastic methods, but then important model realism, such as the sex structure, would be lost. Tahvonen et al. (2018) and Malo et al. (2021) compared optimization results based on the certainty equivalence principle and stochastic optimization. Their results showed that the difference between solutions based on certainty equivalence and stochastic optimization was minor in their bioeconomic models. Thus, in our study, we preferred to maintain model ecological realism and compute the solutions based on the certainty equivalence principle.

## RESULTS

### Analysis of annual reports

Analysis of the annual reports showed that an average 33.5% of winters have been considered somewhat difficult by herders (Table 1). Deep snow cover and icy snow have caused difficulties approximately equally frequently (Table 2). On average, 24.5% of winters have been considered to either have deep snow or icy snow. However, only during an average 8.2% of winters was reindeer mortality considered high or calving success considered poor because of winter conditions, and 4%–5% of winters have been experienced as very difficult (Table 1). Emergency feeding was necessary during these winters, calving success was considered very weak, or mortality was considered very high.

**TABLE 1:** Reported occurrence of above‐average difficult winter conditions in 20 northernmost herding districts in Finland during 30‐year period 1981–2010. The means of the 20 districts are given, along with the minimum and maximum values (percentages [min–max] and numbers [min–max]).

Reported difficulty in winter conditions	Mean (min–max) (%)	Mean (min–max) number
Above‐average difficulties in winter conditions	33.5 (10–50)	10.1 (3–15)
Need for emergency feeding/above‐average feeding needs	3.8 (0–16.7)	1.2 (0–5)
High mortality	5.3 (0–6.7)	1.6 (0–5)
Weak calving success	4.8 (0–20)	1.5 (0–6)
Winters with severe reindeer losses	4 (0–13.3)	1.2 (0–4)
Mortality and/or weak calving	8.17 (0–20)	2.45 (0–6)

Abbreviations: max, maximum; min, minimum.

**TABLE 2:** Reported causes of difficult winter conditions in 20 northernmost herding districts during 30‐year period 1981–2010. The means of the 20 districts are given, along with the minimum and maximum values (percentages [*n*] [min–max] and numbers [min–max]).

Reported causes of difficult winter conditions	Mean (min–max) (%)	Mean (min–max) number
Deep snow	13.7 (0–26.7)	4.1 (0–8)
Icy snow/basal ice	13 (3.3–26.7)	3.9 (1–8)
Late snow melt/late spring	11.2 (3.3–23.3)	3.4 (1–7)
Cold spell	5.3 (0–13.3)	1.6 (0–4)
Cascading effects of previous season/year	1.2 (0–6.7)	0.4 (0–2)
Deep snow or icy snow/basal ice	24.5 (6.7–40)	7.4 (2–12)

Abbreviations: max, maximum; min, minimum.

Supplementary feeding as a coping strategy emerged during the period 1981–2020 in our study districts, as clearly seen in the annual management report references. During the first decade (up to 1990), the 20 northernmost herding districts under study mentioned calf losses and reindeer mortality as general impacts caused by difficult grazing conditions. Impacts on herding practices and supplementary feeding as a specific coping strategy were mentioned only twice. The next decade (up to 2000) saw a significant increase in references mentioning feeding. Coping with the help of supplementary feeding was mentioned in 15 cases; two additional references mentioned feeding in enclosures, and one reference mentioned the intensification of feeding, giving the impression that some supplementary feeding was already happening. Interestingly, only a few references mentioned supplementary feeding during the latest decade (up to 2010). Normalization of the practice could be one reason. Some references mentioned that the feeding period began early because of difficult snow conditions encountered by the reindeer.

The annual reports also mentioned late snowmelt several times, often in connection with a late start of the growing season. Long periods with very low temperatures during winter were mentioned, causing reindeer conditions to decline, along with occasional mention of cascading effects of previous years or seasons (reindeer condition being hampered, e.g., due to a previous difficult summer).

### Dynamic model solutions and convergence to an optimal steady state

**FIGURE 3:**
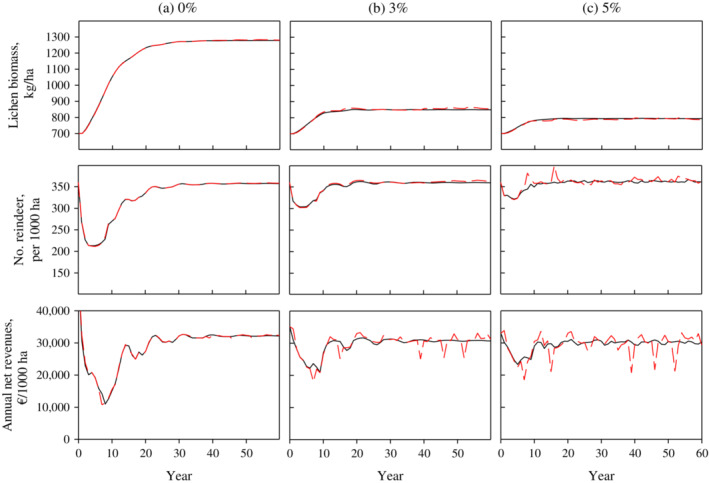
Examples of dynamic economically optimal solutions with 0%, 3%, and 5% interest rates. The black line represents a solution with constant winter conditions (average winters); the red dashed line indicates a solution with stochastic winter conditions (easy, average, and difficult winters vary according to the estimated probabilities [10%, 80%, 10%]).

With the bioeconomic model, we first computed the optimal dynamic solutions with constant average winter conditions (Figure 3, solid black lines). We began from various initial states out of a steady state using 0% to 5% interest rates. Similarly, as demonstrated in Tahvonen et al. (2014), the deterministic model solutions led to a long‐term steady state or to a cycle around the steady state after the transition phase, even if the initial state was far removed from the steady state. The solution type (steady state or cycle around the steady state) depended on the model parameters (e.g., interest rate) and on the linearity of the objective (Pekkarinen et al., 2015; Tahvonen et al., 2014). However, the difference in the present values of net revenues was minor between these solution types with a given interest rate.

We then computed the solutions with stochastic winter conditions assuming that the probability of easy, normal, and difficult winters was 10%, 80%, and 10%, respectively. Figure 3a shows an example of the dynamic solutions using a 0% interest rate with and without stochastic winter conditions. With constant winter conditions, the steady‐state solutions were reached after the transition, but with stochastic winter conditions the system departed from the steady state during difficult and easy winters. However, at a 0% interest rate, the effects of stochastic winter conditions compared to constant winter conditions were very small, especially when lichen biomass was close to the optimal steady‐state level (Figure 3a). Tahvonen et al. (2014) and Pekkarinen et al. (2015) showed that at a 0% interest rate, the steady‐state lichen biomass level was relatively high (typically over 1000 kg/ha). Figure 3a shows that this resource availability is high enough to ensure that survivability and calf production remain high also during difficult winters (excluding extremely difficult winters that were not included in this study).

In addition to the 0% interest rate, Figure 3 shows the dynamic solutions for 3% and 5% interest rates. A higher interest rate implies lower lichen biomass, which makes the system more sensitive to changes in reindeer energy balance during difficult winters. Thus, when the interest rate is higher, and lichen biomass consequently lower, stochastic winter conditions affect the optimal solutions more and clearly differ from solutions with constant winter conditions.

### Slaughtering strategy in optimal steady states

**FIGURE 4:**
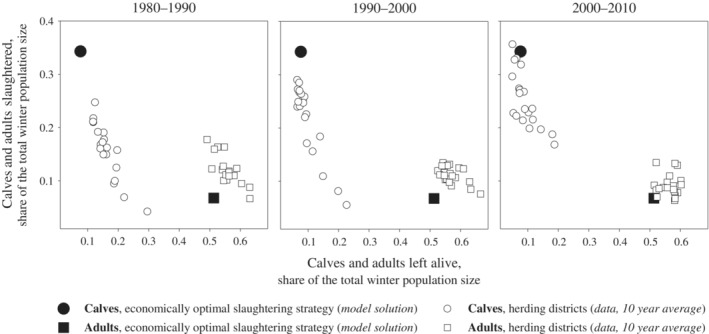
Optimal slaughtering strategy predicted by the model compared with data from the northernmost reindeer herding districts in Finland over three decades during the latest climatological standard period. Black symbols denote model solutions, white symbols represent data. The sum of the shares (live calves + slaughtered calves + live adults + slaughtered adults) represents the total population size just before autumn slaughtering. The comparison shows that the slaughtering strategies in the districts have evolved toward the model solution.

In all optimal steady states found in this study, the slaughtering strategy was based on calf slaughtering. Similarly, as shown by Tahvonen et al. (2014), the optimal slaughtering strategy targets calves, 9‐year‐old females and 5‐year‐old males. At the steady states, more than 95% of male calves and 60% of female calves were slaughtered and the number of males was kept as low as possible while still ensuring a high reproduction rate in the population. Figure 4 compares this optimal slaughtering strategy with data from the northernmost reindeer herding districts in Finland over a three‐decade period. The comparison shows that the actual slaughtering strategies in the herding districts shifted toward the optimal model solution during the previous climatological standard period (1981–2010). During the period 2000–2010, many of the herding districts were already very close to the optimal model solution. The differences between model solutions and data may be partly explained by the model solutions representing a simplified analysis, whereas other factors, including predation pressure, also affect the slaughtering decision in real herding districts (see Pekkarinen et al., 2021 for the effects of predation pressure on optimal slaughtering strategy).

### The cost of a single difficult winter

**FIGURE 5:**
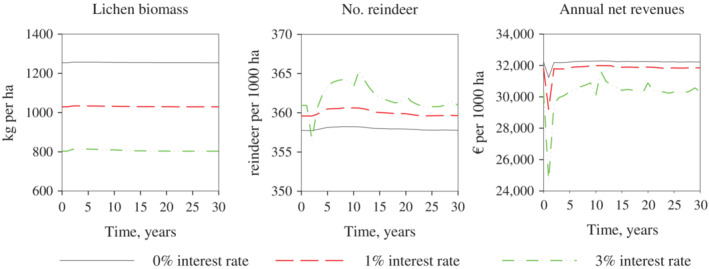
Economically optimal adaptation to a single difficult winter. Optimal solutions computed with 0%, 1%, and 3% interest rates.

Figure 5 and Table 3 show how a single difficult winter affects the economically optimal solutions at different interest rates. The initial states are the economically optimal steady states of the system, with constant average winter conditions for corresponding interest rates. The effects of a single difficult winter on lichen biomass and on the size of the reindeer population are small. Even at a 3% interest rate, the size of the reindeer population decreases by only 1% compared to the steady‐state situation. However, the impact of a difficult winter on the net revenues is clearly higher. With a 3% interest rate, the first‐year net revenues are 19% lower than at the steady state. However, reacting optimally to the effects of these difficult winters increases the net revenues for the following years compared to the steady state (Figure 5 and Table 3). This mainly happens because lichen biomass increases during a difficult winter owing to reduced grazing consumption. Thus, the optimal adaptation and increased lichen biomass clearly reduce the impact of the difficult winter on the reindeer economy. At 3% interest rates, the total discounted future cash flow was 11% lower because of the single difficult winter compared to the discounted cash flow in a constant optimal steady‐state situation. Discounted losses were higher than undiscounted losses (Table 3) because we were studying a situation where the single difficult winter and its main effects occurred during the first year, but the benefits from increased lichen biomass were actualized later.

**TABLE 3:** Costs caused by a single difficult winter under economically optimal adaptation. The initial state of the system before the difficult winter is the economically optimal steady state with the given interest rate and average constant winter conditions.

Annual interest rate (%)	Net revenues	Costs caused by a single difficult winter			
	Optimal steady state	First year	From second year	Total undiscounted	Total discounted
	€/year	€ (%)	€ (%)	€ (%)	€ (%)
0	32,223	990 (3%)	−740 (−2%)	250 (1%)	250 (1%)
1	31,854	2650 (8%)	−1520 (5%)	1130 (4%)	1310 (4%)
3	30,355	5680 (19%)	−3290 (−11%)	2390 (8%)	3470 (11%)

**FIGURE 6:**
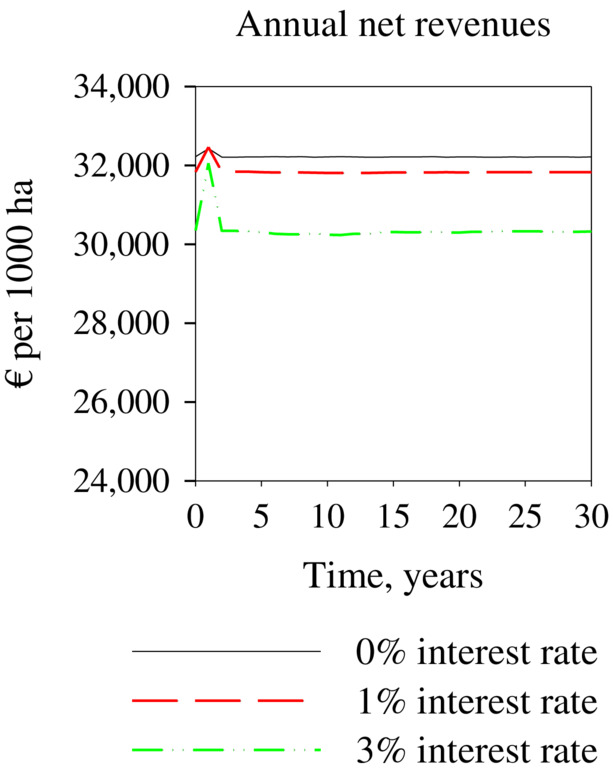
Effect of a single easy winter for annual net revenues from reindeer husbandry in an optimal steady state. Optimal solutions computed with 0%, 1%, and 3% interest rates.

Figure 6 and Table 4 show the effects of a single easy winter for the net revenues of reindeer husbandry. The first‐year increase in net revenues was only 0.7% at a 0% interest rate and 5.6% at a 3% interest rate. Similarly, as with a single difficult winter, the adaptation in following years also diminished the first‐year effect with a single easy winter. Again, this was caused by the delayed effect due to a change in lichen biomass. However, in this case, easy winter conditions caused increased lichen consumption and wastage. This reduced lichen biomass in turn caused a slight decrease in net revenues during following years. The total increase in net revenues due to a single easy winter was 3.6% at a 3% interest rate compared to the steady‐state situation. Thus, the gain from a single easy winter is clearly lower than the loss from a single difficult winter.

**TABLE 4:** Benefits due to a single easy winter under economically optimal adaptation. The initial state of the system before the easy winter is the economically optimal steady state with the given interest rate and average constant winter conditions.

Annual interest rate	Net revenues	Benefits due to a single easy winter			
	Optimal steady state	First year	Second year onwards	Total undiscounted	Total discounted
	€/year	€ (%)	€ (%)	€ (%)	€ (%)
0%	32,223	208 (1%)	−42 (0%)	166 (1%)	166 (1%)
1%	31,854	633 (2%)	−201 (1%)	432 (1%)	446 (1%)
3%	30,355	1704 (6%)	−612 (−2%)	1092 (4%)	1218 (4%)

### The costs of varying winter conditions

**FIGURE 7:**
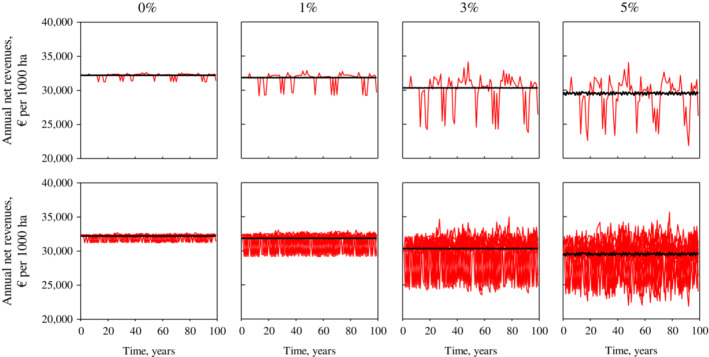
Effects of stochastic winter conditions for reindeer herding economy. Optimal solutions presented for 0%, 1%, 3%, and 5% interest rates. Black lines represent deterministic solutions with constant winter conditions. Red lines show optimal feedback solutions with stochastic winters. Upper panels show example of a single deterministic and single stochastic solution. Lower panels show example of 20 different stochastic solutions with single deterministic solutions.

Next, we computed the net effects of stochastic winter conditions over 100 years using different interest rates. Figure 7 shows optimal deterministic feedback solutions at 0%, 3%, and 5% interest rates for 20 different realizations of stochastic winter conditions. The total number of solutions computed was 50 for each interest rate. In all these solutions, the initial system state corresponded to the deterministic optimal steady state (i.e., constant winter conditions) for the given interest rate. Table 5 gives the present values of net revenues in constant and stochastic winter conditions for a 100 year time horizon at 0%, 1%, 3%, and 5% interest rates. The solutions for stochastic winter conditions were the average net present values of revenues for 100 years over the 50 stochastic solutions. Table 5 shows that even when easy and difficult winter conditions were equally common, stochastic winter conditions reduced average net revenues over a long time horizon. However, the losses from varying winter conditions averaged less than 1% of the present value of net revenues when a reindeer herding district optimally adjusted its slaughtering decisions after untypical winters. During this adaptation, the size and structure of the reindeer population was annually balanced with lichen availability by changing the slaughtering strategy. Figure 5 shows an example of this adaptation, where a difficult winter protected the lichen biomass, allowing the herding district to slightly increase the size of the reindeer population for the following years.

**TABLE 5:** Present values of net revenues (euros per 1000 ha of winter lichen pasture) computed with the reindeer husbandry model for 100 years.

Annual interest rate (%)	Present value of net revenues (€ per 1000 ha)		
	Constant winter conditions	Stochastic winter conditions	Loss due to stochastic winter conditions
	€/100 years	€/100 years	€/100 years (%)
0	3,221,800	3,197,000	2100 (0.1%)
1	2,026,600	2,019,900	6800 (0.3%)
3	987,200	979,000	8200 (0.8%)
5	615,000	609,900	5100 (0.8%)

*Note*: The optimization solutions are presented for constant average winter conditions and for stochastic winter conditions (average over 50 solutions) with interest rates from 0% to 5%. In stochastic solutions, 80% of winters are average, 10% are difficult, and 10% are easy. The final column gives the average loss due to stochastic winter conditions compared to constant winter conditions.

### The costs of a higher frequency of varying winter conditions

**FIGURE 8:**
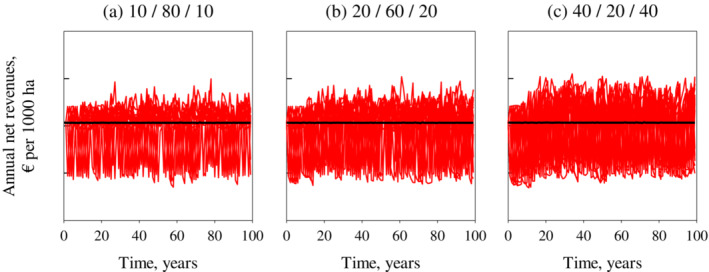
Effects of stochastic winter conditions for reindeer herding economy. Optimal solutions presented for different frequencies of easy and difficult winters. The probabilities of difficult and easy winters are 10%, 20%, and 40% for panels (a), (b), and (c), respectively.

Figure 8 shows 20 different realizations of stochastic winter conditions and optimal feedback solutions at a 3% interest rate and various winter condition frequencies. Increasing the frequency of difficult and easy years increased the losses even when easy winters and difficult winters were equally common. The main reason for this is that the benefit of an easy winter is smaller than the loss caused by a difficult winter (Figures 5 and 6). When 20% of winters were easy, 20% hard, and 60% average (Figure 8b), the annual present value of net revenues was €96,780 (an approximately 2% decrease). However, when the probability of difficult and easy winters increased to 40% (Figure 8c), the average present value of net revenues was €95,510, which was more than a 3% loss, although the slaughtering decisions were optimally adjusted after atypical winters.

### Effects of supplementary feeding

**FIGURE 9:**
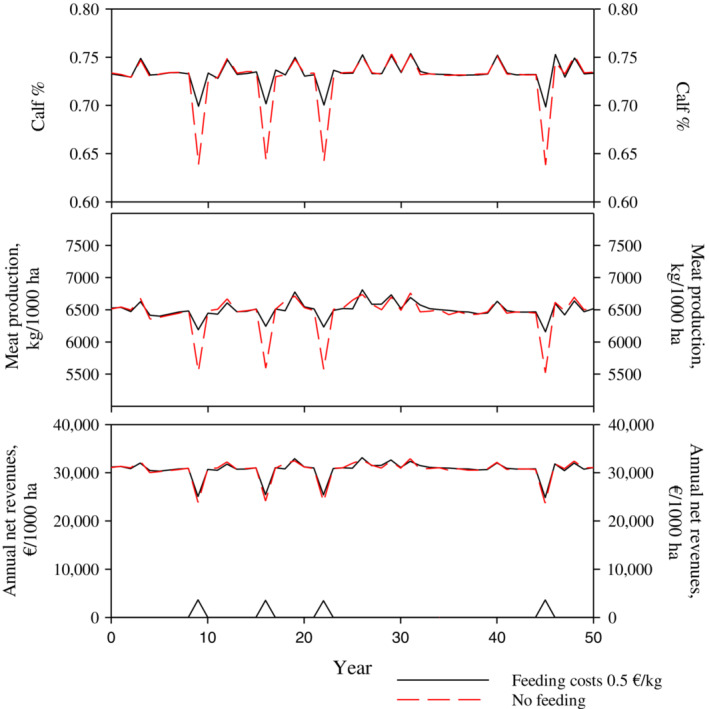
Example of economically optimal solutions with stochastic winter conditions and 3% interest rate. Black lines represent solutions with possibility of supplementary feeding, with feeding costs of €0.5/kg. Red dashed lines represent solutions where providing supplementary food is not possible. The lower lines represent the feeding costs and the upper lines total net revenues.

In all previous solutions, we assumed that supplementary feeding was not available as an adaptation strategy for reindeer husbandry. Figure 9 shows examples of economically optimal dynamic feedback solutions with stochastic winter conditions and a 3% interest rate. Solutions were computed for a case where supplementary feeding was not possible and for a case where the use of supplementary feeding was optimized. The cost of supplementary feeding was assumed to be €0.4/kg of supplementary food delivered to pastures. According to our solutions, at a 3% interest rate, it was not economically sensible to use supplementary feeding during average or easy winters in this type of herding district (a seasonal pasture rotation system is used, winter lichen pastures are located in old or mature pine forests). However, if supplementary feeding is possible and costs €0.4/kg, it is economically sensible to feed reindeer during difficult winters (Figure 9 black line). This leads to a higher calf percentage and meat production. However, because feeding incurs extra costs, the loss in net revenues caused by difficult winters is nearly as high as without the use of Supplementary food.

## DISCUSSION

In this study, the effects of difficult winter conditions on reindeer husbandry were studied by combining economic–ecological model analysis with an analysis of annual reports from Finnish reindeer herding districts. Both analyses suggested that difficult winters affected the productivity and economics of reindeer husbandry. Our analysis of the annual herding district reports showed that difficult winter conditions were frequently experienced by herders. During our study period (1981–2010), 33.5% of winters were perceived as somewhat difficult. Herders reported that above‐average difficult snow conditions occurred during around 25% of all winters. However, survivability or calving success was low only during approximately 8% of the winters due to difficult snow conditions. This result is close to earlier observations made by Helle and Kojola (2008) and Rasmus et al. (2014). Herders also reported that above‐average feeding was used during approximately 4% of the winters due to difficult snow conditions. Extremely difficult winters were experienced approximately once every 25 winters.

Our economic–ecological model structure assumed good summer pastures, but the winter energy gain by reindeer depended on pasture state and winter conditions. This approach was also supported by herder views expressed in annual reports. For example, for herding year 1968–1969, herders from the Käsivarsi district acknowledged: “Summer pastures are sufficient, but the sufficiency of winter pastures greatly depends on the snow conditions,” and herders from Vanttaus district (herding year 1971–1972) observed: “Summer pastures are good. Winter pastures are worse because of icing of the basal layer of snow; reindeer had to be given supplementary feed.” Also, herders from Halla district (herding year 1979–1980) stated: “Pastures sustain the present number of reindeer if snow conditions allow the reindeer to dig.” These excerpts illustrate that pasture use by reindeer, supplementary feeding, and snow conditions must be included in the analysis when analyzing economically sustainable reindeer husbandry.

Using an earlier model version than the one used in this study, Tahvonen et al. (2014) found that the optimal slaughtering strategy was to rely on intensive calf slaughtering and on a low proportion of males in the adult population. Pekkarinen et al. (2015, 2020, 2021) found this solution to be very consistent in the majority of conditions studied with different model versions. However, very high predation pressure (Pekkarinen et al., 2020) or major changes to costs, prices, or government subsidies (Pekkarinen et al., 2021) may change the optimal slaughtering strategy. In this study, we showed that the slaughtering strategy in Finnish reindeer herding districts had clearly evolved during the current climatological standard period (1981–2010) in the direction of the optimal strategy predicted by the model. Pekkarinen et al. (2017) showed that the bioeconomic reindeer husbandry model could describe the dynamics between reindeer population and lichen pastures. Results in this study further verified the applicability of this model by suggesting that the optimization approach used captured features that had been present in the actual slaughtering decisions made by herders.

Our results with the bioeconomic model showed that the typical variation between winters caused economic losses for reindeer herding compared to constant average winter conditions. The net revenues were up to 20% lower during typical difficult winters, mostly because of the lower calf production. However, because lichen biomass was protected from grazing by deep or icy snow cover during these winters, the net revenues in following years were above average and the total loss caused by a single difficult winter was only up to 8%. Herders also expressed this in the annual reports. For example, concerning the winter of 1980–1981, herders from Vätsäri district acknowledged: “The winter pastures were saved from foraging during late winter because of the hard snow cover.” Indeed, this has been part of herder knowledge for decades, as herders from Isosydänmaa district expressed already during winter 1948–1949: “Pastures are good because the reindeer stock has been low; the grazing land has been preserved. In addition to this, lichen has been saved from foraging during two consecutive winters because of snow icing; this has made the foraging of lichen difficult for the reindeer.” These quotes show that local knowledge of reindeer herding includes understanding that the reindeer, pastures, and herders are a system with dynamic interactions and time delays.

In addition to lichen being protected during difficult winters, the net revenues increased during easy winters. Thus, the total difference in the present values of net revenues over long time horizons was small between the optimal solutions in constant and varying winter conditions. Our results therefore suggest that the bioeconomic analysis, which assumed constant average winter conditions, could in many cases also be applied to real‐world situations with typical between‐year variation in winter conditions. However, we did not study the effects of exceptionally difficult winter conditions. In the time of a rapidly changing winter climate, it may be challenging to define what constitutes normal, rare, exceptional, and unexpected winter conditions. Winters we now consider exceptional may become more frequent in the future due to climate change. The warming climate may also increase vascular plant abundance, which leads to a decline in lichen availability (Cornelissen et al., 2001). This decreases the net revenues of reindeer husbandry and increases the negative effects of climate change on reindeer husbandry beyond that found in this study. In addition, lichen biomass was actually lower in most herding districts than in the optimal model solutions presented here. Our solutions suggest that this will further increase the negative effects of difficult winters.

Moxnes et al. (2001) found that typical interest rate levels (0%–5%) only have a minor effect in their biomass model on the optimal size of the reindeer population or lichen biomass. However, in their age‐ and sex‐structured model, Tahvonen et al. (2014) found that increasing the interest rate from 0% to 5% caused a 40% decrease in lichen biomass, although the reindeer population only increased by 5%. This strong effect of the interest rate on lichen biomass was caused by a reindeer's ability to compensate for low lichen biomass with other food plants. When lichen are plentiful, reindeer also consume and waste more lichen than needed to maintain their body in good condition to ensure high calf production and survivability. In this study, we found that because a higher interest rate decreased the optimal steady‐state lichen biomass, the reindeer herding system became more vulnerable to the effects of difficult winters. With a lower lichen biomass, reindeer have a hard time maintaining their bodies in a sufficiently healthy condition during difficult winters due to poor food availability. This happens more easily during extremely difficult winters, which have been estimated to occur approximately four times a century. However, such conditions were not studied here. During these winters, extensive icing locks pastures, leaving reindeer with no way to dig through to the underlying lichen. In those cases, the choices would be either avoiding the ice‐locked pastures (mobility) or providing intensive supplementary feeding.

Pekkarinen et al. (2015) found that in economically optimal steady states, increasing the interest rate could lead to intensive supplementary feeding. They also found that it was economically sensible to use supplementary feeding while recovering from overgrazing. Supplementary feeding has also been used in Finland for decades as a coping strategy during winters with difficult snow conditions (Turunen et al., 2016; Turunen & Vuojala‐Magga, 2014). Also, in this study, we found that herders acknowledged the importance of supplementary feeding as a complementary energy source under conditions of reduced natural winter food. For example, herders from the Hossa‐Irni district (herding year 1982–1983) observed: “Pastures sustain the present number of reindeer, considering the increased feeding in the home enclosures, one third of animals.”

According to herders, difficult winters also increase their workload because they need to increase their control over their herds and intensify supplementary feeding (Turunen et al., 2016). In a study on changing seasonal climate by Rasmus et al. (2020), many herders expressed the view that, although supplementary winter feeding increases expenses, it is currently the only way to ensure regular income from herding. Also, according to our model solutions, it is economically sensible to use supplementary feeding during difficult winters. However, with the estimated current costs of supplementary feeding, the total economic gain remains low and thus difficult winters continue to cause economic losses for reindeer herding even with the use of supplementary feeding. If herders could obtain supplementary food at lower costs, for example, as a result of producing hay from their own fields, the benefit of feeding during difficult winters would be clearly higher than found in this study. In addition, during exceptionally difficult years, feeding may be necessary as the only way to avoid high reindeer mortality.

Reindeer husbandry faces multiple pressures, including climate change, predation, and other land‐use forms (Pape & Löffler, 2012). The model solutions, along with the annual reports presented in this study, show that both economic and ecological factors are important in determining the effects of difficult winters on reindeer husbandry. Results show how pasture conditions, interest rate, supplementary feeding, and the variability of winter conditions all simultaneously determine the optimal herding strategies and the effects on net revenues. However, this is still a simplified picture of the complex net of interactions in reindeer husbandry systems, where pasture conditions, competing land uses, predation, and economic incentives all simultaneously affect reindeer husbandry in addition to the factors included in this study. For example, using the same model setup as in this study, Pekkarinen et al. (2015) showed that intensive supplementary feeding could become the economically optimal solution when the interest rate and government subsidies are high or lichen pasture conditions are poor. In this case, lichen biomass falls to a very low level and supplementary feeding becomes the main energy source for reindeer during winter. This has already happened in many parts of the Finnish reindeer management area. The same bioeconomic model was also used in studying how predation affects the economics of reindeer husbandry (Pekkarinen et al., 2020). A summary by Pekkarinen et al. (2021) concluded that most of the changes in herding conditions in recent decades have had negative effects on the reindeer economy. This study shows that the variation in winter conditions also contributes to these increasing pressures. These pressures reduce the net revenues in reindeer husbandry and simultaneously favor the use of supplementary feeding.

## CONCLUSIONS

In earlier studies, difficult winter conditions were found to increase the mortality and decrease the calving success of reindeer populations. In contrast, during easy winters, calving success and survivability should be higher than during average winters. In this study, we found that, although the net income in reindeer husbandry was higher during easy winters, the losses from difficult winters outweighed these benefits. The effect of difficult winters is especially clear when lichen biomass and other pasture conditions are low due to the high interest rate and intensive grazing or increased effects of forestry and competing land uses.

As also stated by herders in the annual reports, although difficult snow conditions cause economic losses, they also protect pastures from reindeer grazing during these years. Thus, the net income of reindeer herding is higher in the years following a difficult winter. However, according to our economic–ecological model analysis, the overall effect of difficult winter conditions is clearly negative for the reindeer husbandry economy. The use of supplementary feeding increases the capacity of reindeer herding to react to difficult winters, but the current feeding costs are so high that the decrease in net revenues remain almost as high as without supplementary feeding. Lower feeding costs would therefore benefit herders greatly in their efforts to cope with difficult winter conditions.

It has been suggested that climate change has increased the variation in winter conditions of reindeer herding both in amplitude and in frequency (IPCC, 2018; Turunen et al., 2016). In this study, we concentrated on the effect of an increased frequency of varying winter conditions (with both easy and difficult conditions becoming more common). According to our solutions, this increasing frequency of varying winter conditions decreases the income of herders, even if average winter conditions do not change. Thus, our analysis suggests that the increase of variation in winter conditions caused by climate change will negatively affect the reindeer herding economy, even if the harshness of winter conditions during difficult winters does not change.

Combining the use of a state‐of‐the‐art bioeconomic model with practitioner knowledge is rare. Herder knowledge is highly relevant for our research work, and the archive data have seldom used in research. The combination allows for consideration of both scientific knowledge and the practical knowledge of herders, bringing a bottom‐up perspective to the discussion. This study shows that these two very different approaches can be combined and that they validate each other by offering compatible insight, ideas, and understanding that can be accepted by researchers and local communities.

## CONFLICT OF INTEREST

The authors declare no conflict of interest.

## Supporting information


Appendix S1

## Data Availability

AMPL‐code (Pekkarinen, Tahvonen, & Kumpula, 2022) is available on Figshare at https://doi.org/10.6084/m9.figshare.20263905.
