# Supplementary material for: Winter condition variability decreases the economic sustainability of reindeer husbandry
Source: Ecol Appl. 2022 Nov 15;33(1):e2719. doi: 10.1002/eap.2719 (PMC10078097; doi:10.1002/eap.2719)
Supplement: Supplementary file 1 — Appendix S1 [file EAP-33-0-s001.pdf]

Supporting Information for manuscript:

Winter condition variability decreases the economic sustainability of reindeer husbandry

Journal: Ecological Applications

Authors: Antti-Juhani Pekkarinen[1], Sirpa Rasmus, Jouko Kumpula, Olli Tahvonen

[1]Corresponding author

tel.: +358295322529

antti-juhani.pekkarinen@luke.fi

Appendix S1: Model description

The bioeconomic model can be divided into four submodels. The *population model* describes the development of the age- and sex-structured reindeer population. The *energy intake model* computes the daily energy intake of reindeer from various energy resources. The *lichen model* describes the growth, consumption, and wastage of ground lichen. The *economic model* includes prices, costs, and the description of the optimization problem. These submodels are fully presented below but see Tahvonen et al. (2014) and Pekkarinen et al. (2015) for a more thorough description of model details. The full model code is also available for download (Pekkarinen et al. 2022).

Reindeer population model

The population model describes the development of the age- and sex-structured reindeer population. The core of the model is based on classic coupled predator-prey equations but includes much more complexity and endogenous interactions. The main differences to these classic models

are the age and sex structures that can be described with Leslie matrixes for both sexes. The population model also includes descriptions for growth, mortality, and reproduction.

Let $s=0,\ldots,n_f$ denote the female age classes, $x_{0,t}^{f}$ the number of female calves born during the spring of year *t*, and $x_{s,t}^{f}$, $s=1,\ldots n_f, t=-1,0,1,\ldots$ the number of females in each age class at the beginning of each yearly cycle. The yearly cycle in the model begins in autumn after slaughtering. Variables $x_{s,t}^{m}$, $s=0,\ldots n_f, t=-1,0,1,\ldots$ describe the number of males respectively. The number of female and male calves born during spring is given as:

$$x_{0t}^{i}=u_i\sum_{s=2}^{n_f}\beta\left(X_{t-1}^{ef},X_{t-1}^{em}\right)f_s\left(wd_t\right)\left[1-mo_s^f\left(wd_t\right)x_{st}^{f}\right], i=f,m,s=1,\ldots,n_i,t=0,1,\ldots,$$

where u_i, $i=f,m$ denotes the share of female ($i=f$) and male ($i=m$) calves. Female winter mortality is given by $mo_s^f\left(wd_t\right)$, and the average number calves per mated female is given by $f_s\left(wd_t\right)$. Both are presented in Table S1 and are functions of overwinter weight loss wd_t, which in turn is a function of average daily energy intake $\left(E_t^T\right)$ during winter relative to the energy requirement. Average daily energy intake is presented in the energy intake model and depends on winter pasture conditions, and thus is a function of per-hectare lichen biomass (z_t) and the weight (in kg/day) of supplementary food brought to reindeer during winter (v_t). The fraction of females mated at the end of year t-1 is given by the function $\beta\left(X_{t-1}^{ef},X_{t-1}^{em}\right)$, where X_{t-1}^{em} is the "effective number of males" and X_{t-1}^{ef} is the total number of potentially bred females (Table S1).

The numbers of individuals in different age and sex classes evolve according to

$$x_{1,t+1}^{i}=\left(1-m_0\right)x_{0t}^{i}-h_{0t}^{i},\ i=f,m,\ t=0,1,\ldots,$$
$$x_{2,t+1}^{i}=\left[1-m_1^m\left(wd_t\right)\right]x_{1t}^{i}-h_{1t}^{i},\ i=f,m,\ t=0,1,\ldots,$$
$$x_{s+1,t+1}^{i}=\left[1-m_s^i\left(wd_t\right)\right]x_{st}^{i}-h_{st}^{i},\ i=f,m,\ t=0,1,\ldots,\ s=2,\ldots n_i-1,$$

where h_{st}^{i}, $s=0,\ldots n_{i-1}$, $i=f,m$, $t=0,1,\ldots$ denotes the number of harvested reindeer, m_0 denotes constant calf mortality during summer, and $m_s^i\left(wd_t\right)$, $i=f,m$ denotes the adult mortalities (Table

S1). The daily energy requirement for reindeer during winter depends on the weight of the reindeer (age and sex class) and is a function of the difficulty of winter grazing conditions (D_t)

$$Ed_{st}^{i} = \left(1 + 0.06D_t\right)0.683\left(w_{s-1}^{i}\right)^{0.75},\ i = f, m,\ s = 1, \ldots, n_i,\ t = 0, 1, \ldots,$$

where w_s^i is the autumn weight of reindeer in sex class i and in age class s, and D_t gives the difficulty of winter (0=average winter conditions, positive=difficult winter conditions, negative= easy winter conditions). In this study, D_t=1 denotes the conditions during difficult winters and thus the energy need increases by 6% during those winters.

Energy intake model

The energy intake model combined with a lichen model connects the pasture conditions and lichen dynamics to reindeer population dynamics. The energy intake model computes the average daily energy intake $\left(E_t^T\right)$ in winter following the principles of optimal foraging theory. It computes the intake rates for various food sources available to reindeer and selects the combination that gives the highest average energy intake rate. The model takes into account the energy from ground lichens (z_t), other cratered food resources (dwarf shrubs, mosses, and graminoids), and supplementary food (v_t). Lichen biomass develops according to the lichen submodel, but the availabilities of the other cratered food resources are exogenous and constant. The availability of supplementary food is determined by economic optimization.

The average daily energy intake is given as

$$E_t^T = I_t F_t,\ t = 0, 1, \ldots,$$

where I_t is the average intake rate in winter and F_t (Table S1) is the daily foraging time in winter. Both are ultimately functions of lichen biomass (z_t), availability of supplementary food (v_t), difficulty of winter grazing conditions (D_t), and the structure of the reindeer population $\left(\mathbf{x}_{s,t}^{f}, \mathbf{x}_{s,t}^{m}\right)$. If resource availability is low or just adequate to fulfill the energy requirement, the average daily foraging time

is 10h. However, observations show that reindeer energy intake will exceed their energy requirement if there is no shortage of resources. The maximum daily intake in the model is set to 1.4 times the daily energy requirement. With excess resources, reindeer continue to forage even after the energy requirement is fulfilled, although the foraging time begins to slowly decrease. Thus, with high resource availability, reindeer tend to overuse pasture resources during normal or easy winters.

Average intake rate is given as

$$I_t = I_{Zt}\left(1 - T_{Vt}\right) + I_v T_{Vt},$$

where I_{zt} is the intake rate of cratered food items (ie. ground lichens, shrubs, graminoids) and I_v is the constant intake rate of supplementary food. The unit of intake rate is a fraction of the daily energy requirement per foraging hour. Function T_{Vt} (Table S1) gives the share of foraging time used for eating and processing supplementary food. The intake rate of cratered food items is a function of lichen biomass (z_t) and difficulty of winter conditions (D_t), and is given as

$$I_{Zt} = \left(0.55 + 0.00005 z_t\right)\left(3 - 0.4 D_t\right)/3, \ \ t = 0,1,...$$

Thus, if the cratering speed of reindeer during average winters is 3 m^2 per hour, then the average cratering speed is 2.6 m^2 per hour during difficult winters (D_t=1).

Lichen model

The lichen model describes the growth and consumption of the lichen biomass during winter. Lichen biomass at the beginning of model year t is denoted by z_t and develops according to

$$z_{t+1} = z_t - l_t + G\left(z_t - l_t\right), \ \ t = 0,1,...,$$

where function $G(z_t\text{-}l_t)$ gives the annual growth of lichen. The per-hectare annual lichen consumption by the reindeer population is given as

$$l_t = \frac{\sum_{i=f}^{m} \sum_{s=1}^{n_i} l_s^i\left(Ed_{st}^i, E_t^L\right) x_{st}^i}{A}, \ \ i = f,m, \ \ s = 1,...,n_i, \ \ t = 0,1,...,$$

where A is the area (in ha) of winter lichen pastures. The per-reindeer lichen consumption during winter is a function of the age- and sex-class -specific energy requirement and of the average daily energy intake from lichen, and is given as

$$l_{st}^{i} = (1+w)\frac{Ed_{st}^{i}E_{t}^{L}}{10.8}d,\ i = f,m,\ s = 1,...,s_{i},\ t = 0,1,...,$$

where w is lichen wastage during winter (Pekkarinen et al. 2017), d is the length (in days) of the winter season, Ed_{st}^{i} is the daily energy requirement, and E_{t}^{L} denotes the average daily energy intake from lichen (Table S1). Difficult winter conditions decrease lichen consumption because the average daily energy intake from lichen (E_{t}^{L}) relative to the energy requirement decreases. This is caused by the decreased cratering speed and cratering area during difficult winters. The increasing energy need during difficult winters has an opposite effect, as it increases lichen consumption. However, with the estimated parameter values (see chapters 2.4 and 2.5), the effect on cratering speed outweighs the effects on the energy need. Thus, the main effect concerning lichen consumption during a difficult winter compared with a normal or easy winter is that lichen is protected during difficult winters because of the difficult cratering conditions.

Economic model

The objective function is given as:

$$\max_{\left\{h_{st}^{i},v_{t},\ t=0,1,...,\ i=f,m,\ s=0,...,n_{i}\right\}} J = \sum_{t=0}^{\infty}\left(R_{t}-C_{t}\right)^{\alpha}\left(\frac{1}{1+r}\right)^{t},$$

where r is the annual interest rate, $\alpha = 1$ refers to the aim of maximizing the present value of net revenues, and $0 < \alpha < 1$ to the preferences for a smooth annual net income level. The annual revenues (R_t) from slaughtering equal:

$$R_{t} = p\gamma\left[\overline{w}_{0}^{f}\left(wd_{t}\mathbf{x_{t}^{f}}\right)h_{0,t}^{f} + \overline{w}_{0}^{m}\left(wd_{t}\mathbf{x_{t}^{f}}\right)h_{0,t}^{m} + \sum\nolimits_{i=f}^{m}\sum\nolimits_{s=1}^{n_{i}}w_{s}^{i}h_{st}^{i}\right],\ t = 0,1,...,$$

where parameter γ is the fraction of carcass weight and *p* the meat price. $\bar{w}_0^i\left(wd_t\mathbf{x_t^f}\right),\ i=f,m$ are the average autumn weights of calves, which depend on the wintertime weight decrease (wd_t) of adult females and on the age structure of females $\mathbf{x_t^f}=\left[x_{1t}^f,...,x_{n_f t}^f\right]$ (Table SA). Costs depend on the total number of individuals slaughtered ($\sum_{i=f}^{m}\sum_{s=0}^{n_i}h_{s,t}^i$), the total number of individuals in the winter population (X_t), the total land area used by a reindeer herding cooperative (*A*+*K*), and the amount of supplementary food given (v_t):

$$C_t=C_s\sum_{i=f}^{m}\sum_{s=0}^{n_i}h_{st}^i+C_X X_t+C_L\left(A+K\right)+C_V v_t,\ t=0,1,...,$$

where *A*+*K* equals the total land area and $C_{co}, co=S,X,L,V$ denote the associated unit costs. X_t denotes the total number of reindeer at the beginning of the yearly cycle, i.e. $X_t=\sum_{i=f}^{m}\sum_{s=1}^{n_i}x_{st}^i$.

The optimization problem is to choose $h_{st}^i,\ i=f,m,\ s=0,...,n_i,\ t=0,1,...$ and $v_t,\ t=0,1,...$ to maximize *J* subject to the population model, energy intake model, and lichen model, including the non-negativity constraints

$$x_{st}^i\geq 0,\ i=f,m,\ s=1,...,n_i,\ t=0,1,...,$$
$$h_{st}^i\geq 0,\ i=f,m,\ s=0,...,n_i,\ t=0,1,...,$$
$$z_t\geq 0,\ 0,1,...,$$
$$v_t\geq 0,\ 0,1,...$$

The initial levels for the state variables are given

$$x_{s0}^i,\ i=f,m,\ s=1,...,n_i\ \ given$$
$$x_{s,-1}^i\left(1-m_{s,-1}^i\right),\ i=f,m,\ s=1,...,n_i\ \ given$$
$$z_0\ \ given.$$

The difficulty of winter conditions D_t is either given or is randomly selected from the estimated probability distribution.

Table S1: Equations

Variable	Equation
Overwinter mortality *(Tahvonen et al. 2014)*	$mo_t^i = \left[1+\exp\left(\frac{0.36-\sigma_i wd_t}{0.011}\right)\right]^{-0.25}, \ \sigma_f = 1,\ \sigma_m = 1.1$
Total mortality *(Tahvonen et al. 2014)*	$m_{st}^i = \min\left\{1, mo_t^i + ma_s^i\right\},\ s = 1,\dots,n_i,\ i = f,m,\ t = 0,1,\dots$
Overwinter weight decrease *(Pekkarinen et al. 2015)*	$wd_t = 0.5\exp\left[-\exp\left(\frac{E_t^T - 0.72}{0.22}\right)\right],\ t = 0,1,\dots$
Modified harmonic mean mating function *(Bessa-Comes et al. 2010)*	$\beta_t = \min\left\{1, \frac{2X_t^{em}}{X_t^{ef} + X_t^{em}}\right\},\ t = -1,0,1,\dots$
Total number of potentially bred females *(Tahvonen et al. 2014)*	$X_t^{ef} = \sum_{s=1}^{n_f}\left(1-m_s^f\left(wd_t\right)\right)x_{st}^f$
The effective number of males *(Tahvonen et al. 2014)*	$X_t^{em} = \sum_{s=1}^{n_m} fm_s\left(1-m_s^m\left(wd_t\right)\right)x_{st}^m$
Average number of calves per mated female *(Tahvonen et al. 2014)*	$f_{st} = \hat{f}_s\left\{1-\left[1+\exp\left(\frac{0.2715-wd_t}{0.0239}\right)\right]^{-0.1488}\right\}1.2272$
Average daily foraging time in winter *(Pekkarinen et al. 2015)*	$F_t = \min\left\{\frac{1.4}{I_t}, 1.8508+8.1492\left[1+\exp\left(\frac{I_t-0.0953}{0.0013}\right)\right]^{-0.0066}\right\}$
The share of foraging time used for supplementary food *(Pekkarinen et al. 2015)*	$T_{Vt} = \min\left\{\left\{0.3\left[0.2+\frac{0.9}{1+\exp\left(\left(I_{Zt}-1\right)/0.5\right)}\right]\right\}, \left(\frac{1.76v_t}{\sum_{i=f}^{m}\sum_{s=1}^{n_i} x_{st}^i Ed_{st}^i}\right)\right\},$ $i = f,m,\ s = 1,\dots,n_i,\ t = 0,1,\dots$
Lichen growth rate *(Tahvonen et al. 2014)*	$G = -0.7008\left(z_t - l_t\right) + \left(z_t - l_t\right)\left(1+\frac{\left(z_t - l_t\right)}{100.5832}\right)^{-0.0853},\ t = 0,1,\dots$
Daily energy intake from lichen *(Pekkarinen et al. 2015)*	$E_t^L = \left(1-T_{Zt}\right)I_{Zt}F_t\left[0.3621\left(1-e^{-0.0048985z_t}\right)+0.5603\left(1-e^{-0.0015299z_t}\right)\right],$ $t = 0,1,\dots$
Average autumn weight of calves *(Tahvonen et al. 2014)*	$\overline{w}_{0t}^i = \frac{8\sum_{s=2}^{n_f}\left[1-m_t^f\left(wd_t\right)\right]\beta_{t-1}f_s\left(wd_t\right)x_{st}^f wc_{st}^i}{},\ i = f,m,\ t = 0,1,\dots$
The birth weight of calves *(Tahvonen et al. 2014)*	$wc_{st}^i = \alpha^i w_s^c 1.0275\left[1+\exp\left(\frac{wd_t-0.3146}{0.0876}\right)\right]^{-1},$ $i = f,m,\ s = 1,\dots,n_f,\ t = 0,1,\dots$

Table S2: Parameters

Parameter	Unit	Value
n_f, n_m		16, 12
u_f, u_m		0.48, 0.52
$\hat{f}_s, s=1,\ldots,16$	calves /female	0, 0.6, 0.85, 0.9, 0.92, 0.92, 0.92, 0.93, 0.93, 0.85, 0.75, 0.6, 0.4, 0.2, 0.05, 0
$ma_s^m,\ s=1,\ldots,n_m$		0, 0, 0, 0, 0, 0, 0, 0, 0.2, 0.5, 0.8, 1
$ma_s^f,\ s=1,\ldots,n_f$		0, 0, 0, 0, 0, 0, 0, 0, 0, 0, 0, 0.1, 0.2, 0.5, 0.8, 1
m_0		0.02
$w_s^f,\ s=1,\ldots,16$	kg	46, 63, 70, 76, 78, 80, 82, 82, 82, 82, 80, 80, 80, 78, 75, 72, 70
$w_s^m,\ s=1,\ldots,12$	kg	50, 74, 91, 108, 115, 120, 122, 122, 120, 118, 115, 110, 105
I_v		0.568
A, K	ha	1000, 2000
d	days	181
w		0.5
α^f, α^m		1, 1.08
$w_s^c,\ s=1,\ldots,n_f$	kg	4.7, 4.7, 5, 5.4, 6, 6.2, 6.2, 6.2, 6.2, 6, 5.8, 5.6, 5.4, 5, 4.7, 4.7
$fm_s,\ s=1,\ldots,n_m$	females / male	1, 7, 15, 20, 22, 20, 18, 15, 10, 7, 3, 1
C_S	€ / animal	13.35
C_X	€ / animal	39.54
C_L	€ / ha	1.14
C_V	€ / kg	0.4
p	€ / kg	8

References

Bessa-Comes, C., Legendre, S., Clobert, J., (2010). Discrete two-sex models of population dynamics: on modelling the mating function. Oecologica 36, 439-445. https://doi.org/10.1016/j.actao.2010.02.010

Pekkarinen, A. J., Kumpula, J., & Tahvonen, O. (2015). Reindeer management and winter pastures in the presence of supplementary feeding and government subsidies. Ecological Modelling, 312, 256-271. https://doi.org/10.1016/j.ecolmodel.2015.05.030

Pekkarinen A.-J., Kumpula J., Tahvonen O. (2017). Parameterization and validation of an ungulate-pasture model. Ecology and evolution 7(20): 8282-8302. https://doi.org/10.1002/ece3.3358

Pekkarinen, A.-J., Tahvonen, O., & Kumpula, J. (2022). Bioeconomic reindeer husbandry model code for manuscript: Winter condition variability decreases the economic sustainability of reindeer husbandry. Figshare, software. https://doi.org/10.6084/m9.figshare.20263905

Tahvonen, O., Kumpula, J., & Pekkarinen, A. J. (2014). Optimal harvesting of an age-structured, two-sex herbivore–plant system. Ecological modelling, 272, 348-361. https://doi.org/10.1016/j.ecolmodel.2013.09.029
